# Current developments in *Coot* for macromolecular model building of Electron Cryo‐microscopy and Crystallographic Data

**DOI:** 10.1002/pro.3791

**Published:** 2020-03-02

**Authors:** Ana Casañal, Bernhard Lohkamp, Paul Emsley

**Affiliations:** ^1^ MRC Laboratory of Molecular Biology Cambridge Biomedical Campus Cambridge UK; ^2^ Division of Molecular Structural Biology, Department of Medical Biochemistry and Biophysics Karolinska Institutet Stockholm Sweden

**Keywords:** ligands, macromolecular model building, molecular biophysics, real space refinement, rotamers, validation

## Abstract

*Coot* is a tool widely used for model building, refinement, and validation of macromolecular structures. It has been extensively used for crystallography and, more recently, improvements have been introduced to aid in cryo‐EM model building and refinement, as cryo‐EM structures with resolution ranging 2.5–4 A are now routinely available. Model building into these maps can be time‐consuming and requires experience in both biochemistry and building into low‐resolution maps. To simplify and expedite the model building task, and minimize the needed expertise, new tools are being added in *Coot*. Some examples include morphing, Geman‐McClure restraints, full‐chain refinement, and Fourier‐model based residue‐type‐specific Ramachandran restraints. Here, we present the current state‐of‐the‐art in *Coot* usage.

## 1 INTRODUCTION

Model building is an essential step in structural biology that facilitates interpretation of structural data obtained by different methods, including macromolecular crystallography (MX) and electron cryomicroscopy (cryo‐EM). *Coot* is an interactive molecular graphics desktop application that provides an environment where model building and refinement can be used together with validation. Originally, *Coot* was designed for interpretation of MX data, the focus being tools for moving and refining one or a small number of residues, or ligands.^1^, ^2^ The same principles were applicable to model building and refinement of cryo‐EM maps,^3^ and tools in *Coot* have now been expanded to assist building of large macromolecular assemblies into such maps. Furthermore, modern computers now have multiple cores and these have been exploited to extend the range and speed of the *Coot* tools.

Some of the tools that have been significantly used in *Coot*, include C‐alpha baton mode and main chain conversion, automatic finding of alpha helices, beta‐strands and ligands, placing helices and strands, generation of idealized DNA and RNA molecules, real‐space refinement, rigid‐body fit, rotate/translate zone, flip peptides, rotamer tools, and validation tools such as density‐fit analysis, rotamer analysis, and Ramachandran and Kleywegt plots.^2^ The location of these tools within the *Coot* GUI have now been redesigned to make the tools easier to find for novice users. Currently, the catalogue of tools is distributed into menu bars that refer to the type of task to be performed and is shown in alphabetical order. Also, the performance and speed of such tools have been optimized for large macromolecules and maps.

Faster refinement goes hand in hand with the addition of new restraint types. For instance, *Coot* integrates additional restraints for nucleic acids and local distance restraints for cryo‐EM three‐dimensional (3D) reconstructions from other programs such as ProSMART and LIBG.^3^ Newly incorporated restraints into *Coot* include those using a nonharmonic function 4 which augment previous RNA Tools^4^ and RCrane.^5^ These are particularly useful for fitting of domains, chains, or full molecules into cryo‐EM maps.

Other improvements in *Coot* include representation, visualization, and validation of ligands. New features include improved chemical diagrams and 2D representation of geometry outliers for validation. Macromolecular model validation has been a mainstay of *Coot* functionality^2^, ^6^that, in combination with refinement tools and Molprobity analysis,^7^, ^8^ helps to correct the quality of the models. Some of the latest updates to model validation are also described here.

### 1.1 *Fitting domains*

**Figure 1:**
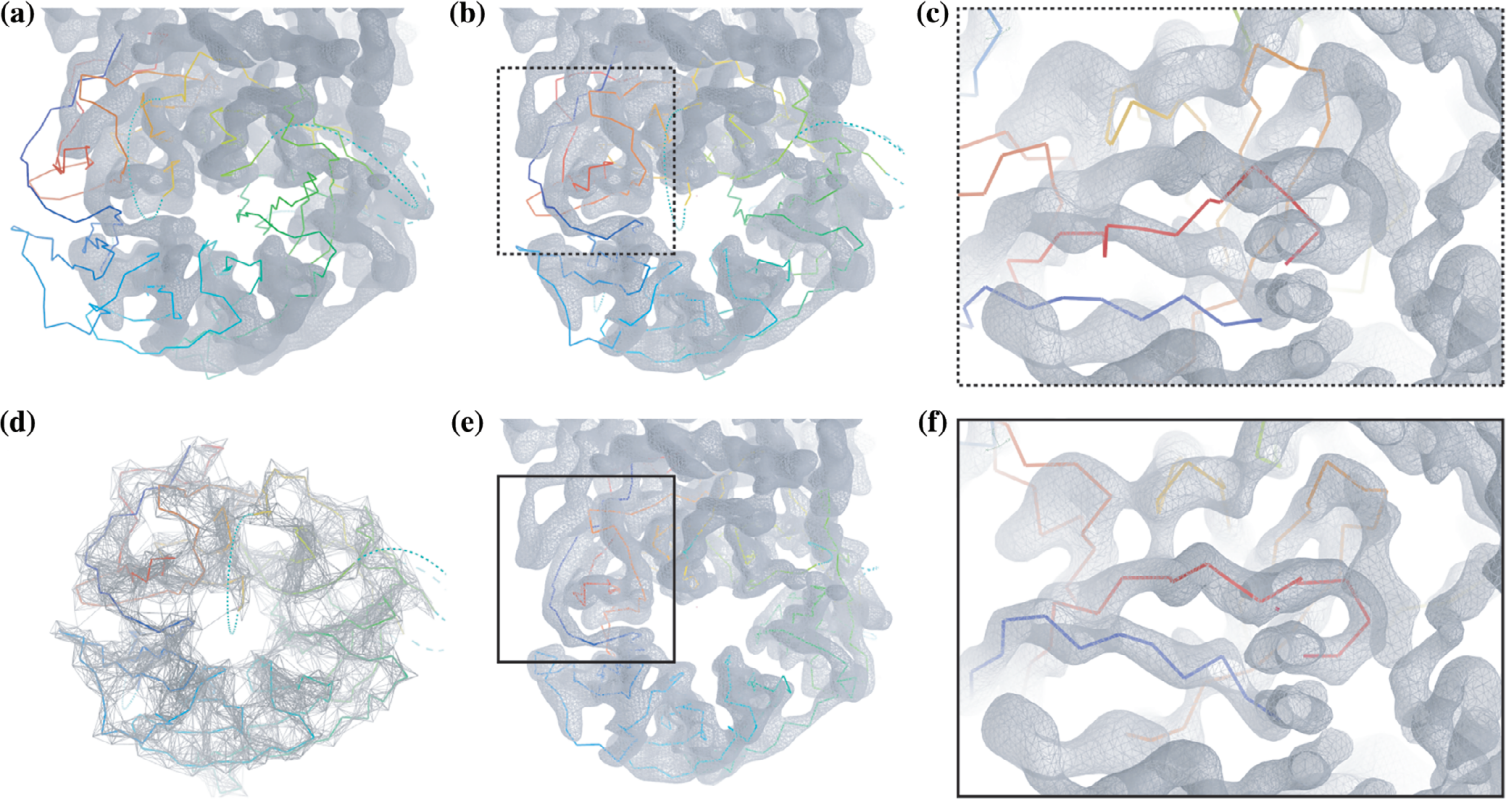
Steps to fit full domains or chains using Coot. After blurring the map (200 A^2^) and placing a homologue near the domain of interest using “move molecule here” (a), jiggle fit (b) improves the fit into the map (c). Geman‐McClure restrains (limit 6 Å) are then applied (d) to perform chain refine (e) to obtain a final accurate fit (f). Map used for representation: EMD‐3908.^44^ Fitted homologue: PDB 6f9n^45^

In cryo‐EM, de novo tracing of the main chains is often needed as the initial step towards structure interpretation. However, if the first operation for map interpretation is to fit the structure of a homolog or a previously obtained model into the map of interest, one would have no need for de novo tracing—just as, in MX, one would not first try to solve a structure with heavy atom derivatives if molecular replacement (MR) was possible.^9^, ^10^ The new developments that have been introduced in *Coot* allow now to place full domains or chains and refine them. The difference is that whereas MR is performing a systematic search of rotations and translations, the tools in *Coot* for cryo‐EM model placement are a local translation and rotation search so that this process is started manually by placing the homolog structure near the center of the domain to be fitted using “Place molecule here.” After the user has performed this operation, *Coot* tools can be used to fit the model into the map. Figure 1 illustrates this process, which includes initial fitting, morphing and refinement, the tools being blurring, jiggle fit, Geman‐McClure restraints, and real‐space refinement (Video 1).

### 1.2 *Blurring and sharpening maps*

Nicholls and coworkers^11^ have previously described the utility of sharpening and blurring cryo‐EM 3D reconstructions. Using a combination of blurred and sharpen maps aids interpretation of main chain and side chains, respectively. Traditionally, this has been performed in programs such as REFMAC or RELION.^12^, ^13^ More sophisticated ways of improving the map are now available such as LocScale or confidence maps that provide an alternative interpretation.^14^, ^15^

In *Coot*, one can generate sharpened and blurred maps using different tools. For MX data, the Sharpen/Blur tool is interactive and is found in the Calculate menu. For cryo‐EM, we would not use the interactive Sharpen/Blur tool because the FFT takes too long for it to be interactive. In this case, a different Sharpen/Blur tool can be found in the cryo‐EM module. Additionally, the cryo‐EM module has a “Multi‐Sharpen” option, which runs Refmac5 to produce an mtz file that contains the coefficients for a number of sharpened and blurred maps. Simply by reopening the generated and saved mtz file, the user can select the preferred blurring/sharpening temperature factor. The module can be installed using Calculate → Modules → Cryo‐EM. When fitting a domain, we find that it is useful to use a large blur factor (e.g., 200 A^2^) before the fitting steps, as blurring allows a larger radius of convergence for the rigid body fit part of the algorithm^1^ (Figure 1a). Furthermore, we have found, somewhat surprisingly perhaps, that resampling the maps output from image processing programs on a finer grid aids visual interpretation.

### 1.3 *Jiggle fit*

Jiggle fit would then be carried out as the next step, typically on a chain or domain as described before.^3^ One can find this tool in the “Modelling” Module, called “Morph.” The algorithm has been updated so that it takes advantage of multiple CPUs to evaluate rotation/translation hypotheses. The best of these are selected and then (again using multiple CPUs) each potential solution undergoes rigid body refinement. The speed of the jiggle fit algorithm scales well with the number of CPUs. The result will be an improved fit of the domain into the density (Figure 1b,c) that will be then further refined using real‐space refinement with local distance restraints.

### 1.4 *Local distance restraint generation*

Macromolecular refinement involves the use of target distances and angles for bond lengths and angles. Other longer‐range restraints such as chiral, torsion, plane, and nonbonded restraints are typically added. At low resolution (and for the majority of cryo‐EM 3D reconstructions), it is useful to add even longer‐range distance restraints to complement these restraints. These restraints might typically encode distance information for hydrogen bonds or conformation of corresponding structures from homologs. When working with *Coot*, the CCP4 program ProSMART would typically be used to generate this additional restraints set although other suites can be used to similar ends^16^, ^17^, ^18^, ^19^, ^20^ with more effort. The generation of self‐local distance restraints has been added into *Coot*. The use of these additional restraints, which tries to keep distance between atoms similar to those present in the starting model, is often what is needed at the first stages of refinement (and sometimes at later stages also). These restraints help the atoms to move in a concerted manner. Typically, the application of restraints in *Coot* has involved the use of harmonic potentials. However, the application of these local distance restraints in *Coot* involves the “Geman McClure” (GM) robust estimator. Nicholls and coworkers explained the value of using GM restraints in protein refinement.^11^ One of the main advantages is that GM restraints stabilize local distances and thereby make the vector by which any atom is shifted more consistent with the shift of neighboring atoms. They menu item to calculate self‐local distance restraints can be found in the “Restraints” module that can be installed from Calculate → Modules → Restraints, similarly, the ProSMART interface can be activated with Calculate → Modules → ProSMART. Nicholls et al.^11^ typically use a distance of 4.2 Å, but as *Coot* refinement has been improved to use multiple CPUs, now one can use longer distances (and hence use more restraints). Therefore, one might use distances of 6 or 7 Å in the local distance restraints generation (Figure 1d). It is our experience with this method that in order to stabilize the refinement and reach the target position/confirmation, models without side chains need longer restraints than those that include them (typically 6 Å). For sophisticated restraints (such as those to a homolog), one would indeed use ProSMART.

### 1.5 *Chain refinement*

The refinement module of *Coot* has been decoupled from the updating of the graphics by running it in its own thread. Consequently, the pressure to only refine small fragments, so that the graphics could update in a timely fashion, has been removed. Thus, more residues can be refined and their representation updated asynchronously rather than forced for every frame. The concomitant changes to the API, and the update of the calculations of the refinement to use multiple threads, considerably improves the ease of use of real‐space refinement, so that it is routine to refine residue selections as large as a domain or chain (Figure 1e, f).

### 1.6 *Merging fragments and domains*

*Coot* is probably used more than any other programs to fragment and patch together molecules in the process of editing molecules. The Copy Molecule (Ctrl‐C), Copy Fragment, Replace Fragment, and Merge Molecules are useful tools here. They can be found under “Edit” in the main menu bar. To illustrate typical operations, we are going to use an example where the “master molecule” has residues 50–100, which need to be adjusted. One would copy out that fragment from the master molecule (Edit → Copy Fragment → Atom selection for fragment “//A/50–100”), “Last Only” in the Display Manager will focus the attention on just that fragment. One can then operate on that fragment using different strategies, such as jiggle‐fit or real‐space refinement. Finally, the fragment will need to be merged back into the master molecule using “Edit → Replace Fragment.” This replaces the position of atoms in the master molecule with those from the fragment. “Merge Molecules” on the other hand will *add* the atoms of the fragment to the master molecule, with new chain identifiers being created if needed.

There is an additional case of merging molecules: that of merging a ligand. It is typical in this case that a new chain identifier is not desired, but instead the chain identifier that matches the chain to which the ligand is begin attached should be used for the ligand. *Coot* uses a proximity check to find the closest chain, and selects a new residue number that is suitably above those of the extant residues.

### 1.7 *Nudge residues*

One of the most difficult problems faced by modelers using cryo‐EM reconstructions are out‐of‐register errors,^21^ which are residues along the chain that are occupying the position that should rather be occupied by neighboring residues (typically out by one or two residues). The identification of these problems is not straightforward, and it is an important aspect of the ISOLDE interface.^22^ The ability to “nudge” residues along a chain has now also been added into *Coot*, allowing quick generation of alternative hypotheses for the register of residues which the user can then inspect visually. Nudge residues can be used from the Cryo‐EM module (Video 2).

### 1.8 *Align and mutate*

Once a domain or chain has been fitted into a map, one might wish to replace the sequence of the homolog that has been refined by the sequence of the protein of interest. For this operation, one can use the alignment tools of *Coot*, which have been augmented by the ability to read in an externally generated FASTA alignment. This allows the user to use a potentially higher quality alignment than that provided by mmdb.^23^ The detected mutations, deletions, and insertions are then applied as before.

### 1.9 *Backrub rotamers*

Although this tool has been available in *Coot* for some time, the method has not been described before. “Backrub Rotamers” is one of the most frequent operations used in *Coot*, that is, fitting side chains. Rotamers are popular, sterically allowed side‐chain conformations (i.e., those frequently observed in structures of the Protein Data Bank [PDB]). Each amino acid type (other they glycine and alanine) has its own set of rotamers which are tabulated in databases.

**Figure 2:**
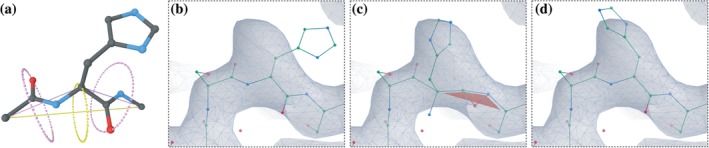
The backrub method. (a) Schematic representation of the backrub motion. The central residue and adjacent peptides move around backrub vector (yellow circle and axes). Individual adjacent peptides back‐rotate around the peptide vectors (pink). This motion preserves the geometry of the main chain while fitting the side chains into the map correctly. The algorithm in action in *Coot* is illustrated in panels (b), (c), and (d). In (c), the backrub option is inactive and the main chain is heavily distorted (appearance of red flags indicate that a *cis*‐peptide has been introduced). When the backrub option is active in *Coot*, the side chain is suitably fitted (d). Map and model used for representation: EMD‐3908, PDB 6eoj^44^

“Backrubs” were introduced into the lexicon of protein modeling operations by Richardson and colleagues.^24^ In this work, the authors aimed to find low occupancy rotamers in high‐resolution maps. In *Coot*, the same formalism for atom movement has been reworked to find high probability rotamers in low‐resolution maps. The previous rigid‐body‐fit‐based rotamer fitting tool in *Coot* allowed poor/impossible backbone geometry in low‐resolution maps, frequently resulted in distorted main chains (Figure 2).

The backrub vector, for any given residue, is the vector between the C‐alpha atoms of the previous and next residues (Figure 2a). The motion of the atoms in backrub fitting is primarily a rotation around the backrub vector, and those moving atoms consist of the atoms of the given residue, the C and O atoms of the previous residue and the N of the next residue. The yellow arc in Figure 2a represents the path (along which hypothetical structures are generated) of the central C‐α atom; however, all the moving atoms rotate about the backrub vector (these arcs/circles are omitted for clarity) (Video 3).

Moving the atoms in such a way generates a number of hypotheses for the position and orientation of C‐α and C‐β of the given residue. Using internal coordinates, the χ‐angles of the rotamers of the type of the given residue provide positions for the rest of the atoms of the side chain. Given these atom positions, the fit to density is assessed, as are any clash interactions with residues in the environment (symmetry atoms are not considered). The score from the clash interactions and the density fit are combined for each hypothesis, and the best hypothesis is selected and replaces the current model if the score for that hypothesis is better than the score for the current positions of the atoms of the given residue. There is, as yet, no secondary structure dependence on the rotamer selection.

The backrub motion introduces a change in τ (the N‐CA‐C angle) typically less than 2°, so while this change may be of some (negative) consequence, it is highly likely to be less than the beneficial changes in φ, ψ, or χs of the fitted residue and the φ and ψ of the neighboring residues. Any introduced τ angle strain can easily be reduced or removed by subsequent refinement (real space or otherwise).

### 1.10 *Ligand fitting and refinement*

#### 1.10.1 *DNA–RNA*

*Coot* has the ability to generate A or B forms of RNA and DNA. These molecular fragments often provide useful templates for de novo model building in cryo‐EM 3D reconstructions. In addition to the standard molecular restraints (bonds, angles, chirals, planes, torsions, and nonbonded contacts),^1^ the additional restraints, to which we refer above, that is, nucleic acid packing and stacking,^3^ and local distance restraints, allow us to fit nucleic acids in even low resolution maps (below 4 Å) allowing the molecules to bend and twist without breaking the stacking interactions or the base pair hydrogen bonds (Video 4).

### 1.11 *Asparagine‐linked carbohydrate fitting*

Asparagine (Asn)‐linked glycosylation is the most common type of N‐glycosylation of eukaryotic proteins, and it is also found in viruses, including HIV and Ebola.^25^, ^26^, ^27^ Agirre reports that the fraction of Asn‐linked glycosylation in the PDB as of 2013 is 5.5% (and increasing).^27^ Until recently, it was difficult for structural biologists to build high‐quality models of carbohydrates since the software tools were not developed for this end.^28^ This combined with the inherent complexity of carbohydrate structure^27^, ^29^ has led to the deposition of carbohydrate structures with errors.^28^, ^30^, ^31^ To assist in this matter, an N‐linked carbohydrate fitting tool has been added into *Coot*.^32^ Now, the restraints and links for this fitting have been augmented with the addition of consensus local distance restraints, care for anomericity in link detection, pseudopartial pyranose ring plane restraints, and unimodal pyranose ring torsion restraints, to stabilize the model in a manner consistent with biochemical principles and prior structural knowledge. The tool has two major modes, guided and automated. The user needs to identify the type of glycan to be built and thus restrict the type and the link type of the carbohydrate monomer to be added. The built‐in glycosylation comprehension then allows the user to select only particular carbohydrate types with particular links. The tools typically generate glycans within a minute with little user intervention.

It should be noted that the density of N‐linked carbohydrate in cryo‐EM reconstructions is often of lower quality that that from MX data. In cryo‐EM, we need to rely more on prior knowledge than optimization of the fit to density. Additional work is needed in *Coot* to bring model building of carbohydrates to a stage where it can routinely produce high‐quality glycan models with cryo‐EM 3D reconstructions.

### 1.12 *JED‐Flip: Hybridization‐aware rotatable bond rotator*

For some time now, *Coot* has had a means to rotate residues and other molecular fragments around rotatable bonds. More recently, a faster tool has been introduced that is particularly useful for ligands. Consider the case in which a ligand is placed in the active site of a protein, with a conformation that is not correct. The ligand has a benzene ring with a chlorine substituted at the meta position and the real/correct conformation needs the benzene ring of the ligand, and its substituents, to be rotated by 180°. Although the “Edit Chi‐angles” tool can do this, the manipulation is faster and more convenient with “JED Flip,” particularly with “Interactive JED Flip,” where the rotation is applied to the “active” bond during refinement. JED Flip uses the torsion restraint to create a number of torsion angle deltas (typically, +/− 120° or 180°), the application of which is frequently the operation needed to correct the conformation of the ligand. If Interactive JED Flip is used, the ligand settles into the correct conformation with no additional intervention (see video). By default, *Coot* moves the smaller set of atoms on either side of the rotatable bond. (Infrequently) one might wish to rotate the larger fragment and this is achieved by “Reverse JED‐Flip” (Shift G) (Video 5).

### 1.13 *Acedrg Interface and links*

Acedrg^33^ is a software for the generation of restraints for compounds for macromolecular refinement. The input to Acedrg is an MDL Mol file, a SMILES string, or a chemical component dictionary. While Acedrg is a general‐purpose restraints generator, as the time of writing, it is not able to generate restraints for compounds that contain metal atoms. Recently, Acedrg has been extended to enable the generation of link restraints (a link being a covalent or other chemical bonds) between compounds. Standard link restraints such as polymer link restraints, phosphodiester link restraints, and disulfide bond links are already in the standard CCP4 Restraints library. The new mode of Acedrg allows the generation of bespoke chemical links, for example, novel amino acid modification or a covalent link between a ligand and a residue of the active site. *Coot* has been extended to provide an interface to this functionality. Using the CCP4 Module (Calculate → Modules → CCP4), one can select an atom in each of two residues and define a bond order (default “single”) which then constructs the input for Acedrg. Acedrg is run in the background and then *Coot* loads the dictionary produced by Acedrg so that it becomes available for Real‐Space Refinement.

### 1.14 *Metal link restraints*

The new version of *Coot* allows the refinement of metals. Previous version of *Coot* did not interpret the LINK records for metals in macromolecules and hence selecting a metal atom in Real‐Space Refinement meant that there was only a nonbonded contact restraints between the metal and the metal ligand atoms. Now, *Coot* parses the LINK records and generates bond restraints for metal between nitrogen, oxygen, and sulfur metal ligands. Furthermore, with the addition of this bond restraint, the nonbonded contact restraint is no longer used. The target distances used for these bond restraints are element based derived from the metals in the Acedrg tables (full‐atom Acedrg atom types are not used so the restraints are not as accurate as they might be in the future).

### 1.15 *Representation and visualization*

#### 1.15.1 *Ligands: Lidia*

The “Ligand Display and Analysis” (Lidia), the framework for 2D representation of ligands, has been previously described.^34^ Since then, the chemical diagrams have been improved. Using Lidia (Draw → Ligand Builder), one can sketch chemical diagrams in the manor of Chemdraw or JMSE.^35^ Having completed the depiction, the “Apply” button will generate a 3D model using a number of dictionary‐generating software/programs and then display that representation in the main graphics window. 3D to 2D is a more straightforward proposition and has been included into *Coot* by wrapping the functions of the RDKit (Open‐source cheminformatics; http://www.rdkit.org/). As well as de novo sketching, Lidia also supports the import of chemical structures from mol or mmcif files, a SMILES string, has a rather robust “Fetch” tool which uses Wikipedia to convert potentially common molecule names to the International Nomenclature Name, and parses the drug box to import a representation of the ligand from DrugBank,^36^ ChEMBL,^37^ ChemSpider (hhttps://www.chemspider.com/Chemical-Structure.1906.html), or PubChem.^38^

Clarke and Labute^39^ describe a method for the 2D depiction of protein‐ligand complexes. This has been implemented in *Coot* using the RDKit for the 2D layout of the chemical entity and adds rendering and representation of protein residues and interactions in the “Flatland Ligand Environment View” or FLEV mode (Ligand → FLEV this residue).^34^ Many of these molecular details are available as pythonic representations using the pli module.

### 1.16 *Maps*

In the past, small fragments of a map would be visualized in the *Coot* graphic interface to proceed with model building and speed up the visualization process. Currently, as a result of multithreaded contouring, one can display larger areas of the working map as well as provide different view styles for the maps, that is, not only the traditional Standard Lines but also Solid/Transparent and Cut‐Glass representations. All of the above together facilitate map interpretation. These options are available under Display Control → Properties.

### 1.17 *“Blob” navigation*

Perhaps, the most convenient but overlooked feature of *Coot* is using the map for navigation. When model building with *Coot*, one wants to add or move atoms to (or close to) the view rotation centre. The reason is that this way allows easier visualization of what is around a particular point if the point is at (or close to) the view rotation centre.

One can do this quickly and easily navigate to the area of interest using the function blob_under_pointer_to_screen_centre() bound to a key‐press (typically “G”). *Coot* determines the mapping into 3D space of the point under the cursor on both the front and back clipping planes, which gives us two 3D points in the model coordinates system. *Coot* then (conceptually) draws a line between these two points and steps, in small increments, from the front 3D point to the back 3D point. As it does so, it queries the value of the active (fitting) map at each of those points and considers that value in relation to the contour level. When it finds a point above the contour level, it starts to record a density profile, and continues until it finds a point below the contour level. The weighted mean position of that profile then becomes the new rotation centre.

### 1.18 *Validation*

Validation of structures has been an important aspect of macromolecular model building for a number of years—after it had been made apparent that it was possible to incorporate both gross and small errors in protein models.

An important aspect of good macromolecular modeling tools is not only to detect problems in the structures but also to provide means to resolve them. The interactive validation tools of *Coot* go in this direction, and it is an on‐going topic of research.

*Coot* currently incorporates an extensive validation menu to assess the model geometry and the fit of the models to the maps. The most practical quality indicators are the Geometry analysis, Difference Map Peaks, Density‐fit analysis, Rotamer analysis, and Ramachandran and Kleywegt plots.^2^ These tools identify problematic regions in the model and allow fast navigation for their resolution. Additional tools for ligand validation have been introduced.^6^ Here, we highlight recent updates.

### 1.19 *Rotamers*

The Rotamer validation has been updated. *Coot* now uses “The (son of) Penultimate Rotamer Library”^40^ to provide the probabilities for the side chains of the protein of interest. These probabilities can be viewed in a validation graph using the Validation menu (Validate → Rotamer analysis) or as the function score_rotamers in the API (Calculate → Scripting → Python).

### 1.20 *Temperature factors*

*Coot* provides basic statistics for model atomic displacement parameters, called “temperature factors” in the *Coot* interface—an analysis of temperature factors can quickly direct the user to parts of the structure that have been erroneously modeled (or are more flexible than the majority of the structure). Interactive display of the temperature factor graph is available in the validation menu. The python functions to return the average and median temperature factors are mean_b_factor(imol) and median_b_factor(imol). The median temperature factor is often a more robust metric than the mean as it will not be affected by a small number of waters that have very high temperature factors.

### 1.21 *Ramachandran plot*

**Figure 3:**
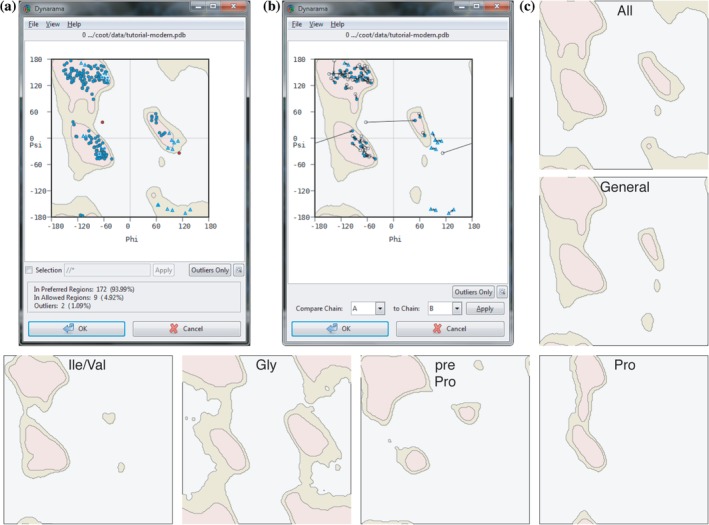
Ramachandran plots in *Coot*. (a) and (b) Ramachandran window in Coot. (a) Ramachandran plot. Each residue of the selected molecule is plotted on the canvas with the preferred (salmon), allowed (beige), and outlier (grey) regions plotted in the background. A selection box below the plot allows display of a selection of residues only. The “Outlier Only” buttons toggle to display all residues or outliers only. Below the plot, the statistics are shown. A menu bar allows, for example, printing of the plot, changing to Kleywegt plot (see b). (b) Kleywegt plot.^42^ Distances between NCS‐related residues are shown in the Ramachandran plot as in (a). The chains to be compared can be selected and the plot updates accordingly. (c) The different Ramachandran plots currently used for backbone validation in Coot: all, general, isoleucine/valine (Ile/Val), glycine (Gly), pre‐proline (pre‐Pro), and Proline (Pro). Colors represent same features as described in (a) and (b)

The Ramachandran plot tool in *Coot* has been updated and improved in various ways. In general, the tool can be launched from the validation menu and will show the Ramachandran plot in a new window (Figure 3). A marker for each residue is plotted on the canvas based on the angle values of the backbone. All residues, except glycine (Gly) and proline (Pro), are plotted as circles; Gly are shown as triangles; and Pro as squares. Residues in allowed and preferred regions are coloured blue; outliers are shown in red. The background shows the preferred, allowed, and disallowed regions of the Ramachandran plot according to the amino acid type. The new plots are based on the top 8,000 library^8^ build into the Clipper library^41^ which differentiates between the following residues: all residues, general‐case (16 amino acids), isoleucine/valine (Ile/Val), Gly, Pro, and pre‐Proline (pre‐Pro) (Figure 3c). The background canvas changes dynamically to show the corresponding plot for the currently selected residue. Contour lines and different color allow the visual distinction between the different areas of the plot. The plots are precalculated images for speed but the grid sampling and cut‐off region is customizable. Furthermore, the new Ramachandran plot allows only a selection of residues to be displayed. Only residues labeled as outliers can be shown by toggling the “Outlier Only” button, and an optional entry widget allows residue selection using CCP4 atom selection syntax. To compare NCS‐related chains in the context of the Ramachandran plot, the Kleywegt plot can be used (Figure 3b), where NCS‐related residues from two chains are plotted on the Ramachandran plot connected by an arrow.^42^ By default, the 50 most distant residues are plotted. In the new interface, the user can switch between these two modes (Ramachandran and Kleywegt) within the same dialog window using a menu button. Furthermore, the selection of chains for the Kleywegt plot can conveniently be done directly in the window for a given molecule or using comparing chains between different molecules, for instance, before and after refinement. The Ramachandran plot widget is also available as a stand‐alone application, *Dynarama*. This way users can benefit from this validation tool and its features outside of *Coot*. The Ramachandran plot widget allows exporting of the plot together with the statistics in pdf and png format. In the future, the Ramachandran plot is envisaged to be even more interactive, for example, to update during the refinement, allow dragging, and flipping of residues within the plot, as well as contain gradient colors, in addition to the contour lines, to allow better visualization of the different regions.

### 1.22 *Ligands*

The idea of the ligand validation tool^6^ was to compare (a) the correlation of the density of the ligand calculated from the model with various maps (including an omit map) and (b) the distortion of the built ligand with other ligands in the PDB (both at similar resolution and overall). However, this tool has not been widely adopted by the community, perhaps because of its dependency on CCDC's Mogul validation program.^43^ Consequently, the validation feedback has been reworked so that it is based on a dictionary rather than using Mogul and so that the module has no external dependency other than Refmac. This functionality can be found in the Ligand → Ligand Metric Sliders.

### 1.23 *Future*

The development of *Coot* will progress in two major directions.

Although writing multithreaded code is difficult, the pay‐off is substantial. Larger atoms selections can be refined faster and with more restraints. Rotation/translation searches, torsion angle searches, and dynamic atom contact searches can be performed using multiple threads to reduce the wait‐time and increase interactivity.

Secondly, a wholesale reworking of the dependencies will provide Python 3, more numerous and more fully featured modules, and an updated GUI which will eventually lead to graphics that are more interactive and better represent molecule shape of both the atoms models and maps.

The combination of both the above will allow for interactive representation of rotamer probability, the Ramachandran plot, clashes, and other validation criteria.

### 1.24 *Access to software*

At the time of writing, the Coot web page is at https://www2.mrc-lmb.cam.ac.uk/personal/pemsley/coot/.

The software is licenced under the GNU GPL v3, GNU LGPL v3, and compatible licences and is available for free for academics and others. Links to source tar files and binary tar files is available from the *Coot* web page. The source code repository is available at https://github.com/pemsley/coot.

## CONFLICT OF INTEREST

The authors declare no conflicts of interest.

## Supporting information

Video 1 Fitting domains, using additional local distance restraints to refine a domain into a cryo‐EM map.

Video 2 Shuffle‐along/Nudge residues–how the active residue selection and be shuffled along the change using locale distance restraints.

Video 3 Backrub Rotamers, a rotation illustrating the ways in which atoms can move to generate hypotheses in Backrub Rotamer fitting.

Video 4 RNA Fitting‐Domains: How to fit a fragment of RNA into a cryo‐EM reconstruction, with narration.

Video 5 JED‐Flip: Example of how a ligand can be quickly and easily rotated by chemically sensible angle differences to interactively examine the fit to density.
